# Disease-Causing Mutations in *BEST1* Gene Are Associated with Altered Sorting of Bestrophin-1 Protein

**DOI:** 10.3390/ijms140715121

**Published:** 2013-07-22

**Authors:** Jordan A. Doumanov, Christina Zeitz, Paloma Dominguez Gimenez, Isabelle Audo, Abhay Krishna, Giovanna Alfano, Maria Luz Bellido Diaz, Veselina Moskova-Doumanova, Marie-Elise Lancelot, José-Alain Sahel, Emeline F. Nandrot, Shomi S. Bhattacharya

**Affiliations:** 1 Biological Faculty, Sofia University “Saint Kliment Ohridski”, 8 Dragan Tzankov str, Sofia 1164, Bulgaria; E-Mail: moskova@biofac.uni-sofia.bg; 2 Institut National de la Santé et de la Recherche Médicale (INSERM), UMR_S 968, Paris F-75012, France; E-Mails: christina.zeitz@inserm.fr (C.Z.); isabelle.audo@inserm.fr (I.A.); marie-elise.lancelot@inserm.fr (M.-E.L.); j.sahel@gmail.com (J.-A.S.); emeline.nandrot@inserm.fr (E.F.N.); smbcssb@ucl.ac.uk (S.S.B.); 3 Centre National de la Recherche Scientifique (CNRS), UMR_7210, Paris F-75012, France; 4 Centre de Recherche Institut de la Vision, Université Pierre et Marie Curie-Paris 6, 17 rue Moreau, Paris F-75012, France; 5 Andalusian Center of Molecular Biology and Regenerative Medicine, Centro Andaluz de Biología Molecular y Medicina Regenerativa (CABIMER), Avda. Americo Vespucio s/n, Parque Cientifico y Tecnologico, Isla de la Cartuja 41092, Sevilla, Spain; E-Mails: paloma.dominguez@cabimer.es (P.D.G.); abhay.krishan@cabimer.es (A.K.); marialuz.bellido@cabimer.es (M.L.B.D.); 6 Centre de Référence Maladies Rares/Centre d’Investigation Clinique (CMR/CIC), 503 INSERM, CHNO des Quinze-Vingts, Paris F-75012, France; 7 Institute of Ophthalmology, University College London, 11-43 Bath Street, London EC1V 9EL, UK; 8 Fondation Ophtalmologique Adolphe de Rothschild, Paris F-75019, France

**Keywords:** BVMD, Best1 protein, cell polarity, MDCK cells

## Abstract

Mutations in *BEST1* gene, encoding the bestrophin-1 (Best1) protein are associated with macular dystrophies. Best1 is predominantly expressed in the retinal pigment epithelium (RPE), and is inserted in its basolateral membrane. We investigated the cellular localization in polarized MDCKII cells of disease-associated Best1 mutant proteins to study specific sorting motifs of Best1. Real-time PCR and western blots for endogenous expression of *BEST1* in MDCK cells were performed. Best1 mutant constructs were generated using site-directed mutagenesis and transfected in MDCK cells. For protein sorting, confocal microscopy studies, biotinylation assays and statistical methods for quantification of mislocalization were used. Analysis of endogenous expression of *BEST1* in MDCK cells revealed the presence of *BEST1* transcript but no protein. Confocal microscopy and quantitative analyses indicate that transfected normal human Best1 displays a basolateral localization in MDCK cells, while cell sorting of several Best1 mutants (Y85H, Q96R, L100R, Y227N, Y227E) was altered. In contrast to constitutively active Y227E, constitutively inactive Y227F Best1 mutant localized basolaterally similar to the normal Best1 protein. Our data suggest that at least three basolateral sorting motifs might be implicated in proper Best1 basolateral localization. In addition, non-phosphorylated tyrosine 227 could play a role for basolateral delivery.

## 1. Introduction

Best vitelliform macular dystrophy (BVMD) is an autosomal dominant inherited disorder that affects central vision starting generally around teenage years [1]. Histopathologically, BVMD has been shown to manifest as a generalized retinal pigment epithelium (RPE) abnormality associated with lipofuscin accumulation, regions of geographic atrophy, and deposition of abnormal fibrillar material beneath the RPE [2]. In addition, patients are also diagnosed with a decreased electrooculogram (EOG) [3,4]. BVMD is linked to mutations in the *Bestrophin-1* gene (*BEST1*, formerly *VMD2*) located on chromosome 11 [5,6]. Mutations in *BEST1* are associated with phenotypic heterogeneity and cause five clinically distinct human diseases—the classical BVMD, adult-onset vitelliform macular dystrophy (AVMD), autosomal dominant vitreoretinochoroidopathy (ADVIRC), autosomal recessive bestrophinopathy (ARB) [7] and retinitis pigmentosa (RP) [8–10]. The *BEST1* gene encodes the 585-amino acid protein bestrophin-1 (Best1) with an expected molecular mass of 68 kDa. The protein is expressed predominantly in the RPE and localizes mostly to the basolateral plasma membrane of the cells [11]. Two different models suggest that Best1 is a transmembrane protein with four transmembrane domains; its amino and carboxyl termini are localized within the cytoplasm, and the protein is not modified by N-linked glycosylation [12,13]. The main difference between these two models concerns the arrangement of transmembrane domains (TMDs) four and five in the lipid bilayer of RPE cells.

Experimental data suggest that Best1 proteins are involved in Ca^2+^-dependent transport of chloride ions across cellular membranes as Cl^−^ channels [14,15]. The protein is involved in the signal transduction pathway that modulates the light peak of the EOG, which might be regulated by phosphorylation of bestrophin-1 [16]. The hallmark diagnostic feature of BVMD is a decreased or absent slow light peak noted when measuring EOG [4,17]. Sun and colleagues proposed that this peak reflects a Cl^−^ conductance in the basolateral membrane of the RPE [14,18]. Marmorstein and colleagues suggested that human Best1 affects the light peak indirectly via its regulation on voltage-gated Ca^2+^ channels [19,20]. *Best1* knock-out (*Best1*^−/−^) mice exhibit a normal or enhanced light peak, therefore Best1 might not be required for generating the light peak but might serve to antagonize it [21]. In addition, it was reported that bestrophin-1 protein conducts HCO^−^_3_ similar to Cl^−^[22] and mediates glutamate release in astrocytes [23,24].

Polarity of RPE is crucial in maintaining homeostasis of the whole retina. In polarized epithelial cells the plasma membrane is divided into apical and basolateral domains, separated by tight junctions and having distinct compositions of proteins and lipids that are exposed to different environments [25–27]. Polarity is particularly important for channels that specifically transport ions and other molecules at the basolateral or apical domains. Hence, the proper basolateral localization of Best1 is important for its function in RPE cells. Indeed, Mullins and co-workers showed that in a BVMD patient with a p.Y227N mutation, the Best1 protein localized not only along the basolateral membrane but also in the apical membrane of the RPE [28].

Basolateral and apical protein targeting depends on specific motifs in the protein structure. Basolateral delivery is usually mediated by specific signals located in the cytoplasmic domains of proteins. These signals often involve tyrosine-based (YXXØ, where Ø is an amino acid with a bulky hydrophobic side chain and X stands for variable amino acids) or di-leucine (LL) motifs [29,30]. Best1 displays several potential basolateral sorting motifs, e.g., Y85VTL, Y97ENL, L206L207 and Y227DWI. In contrast, apical targeting has been attributed to different types of sorting signals, including N-linked or O-linked carbohydrates, specific transmembrane domains, GPI anchors or cytoplasmic domain determinants [31,32].

In the present study expression constructs of several disease-causing mutations (p.Y85H, p.L100R, p.L207I and p.Y227N) identified previously [6,33,34] and disrupting putative sorting motifs of Best1 were generated. We then analyzed the mutants in respect to their basolateral targeting in polarized MDCK cells, a trusted and widely-used model for epithelial cell polarity studies [35,36]. Additionally, we analyzed the localization of one new mutation identified in our cohort that has not been described previously (c.287A > G; p.Q96R).

## 2. Results and Discussion

### 2.1. Expression of Bestrophin-1 in MDCK Cells

**Figure 1:**
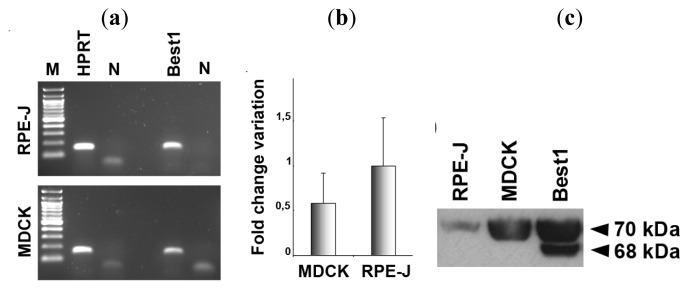
(**a**) *HPRT* and *BEST1* mRNAs are expressed in MDCK and RPE-J cells. M–100 bp ladder, N–negative control; (**b**) Quantification of *BEST1* expression levels between MDCK and RPE-J cells using quantitative Real-Time PCR. Fold change variation in *BEST1* expression levels is reported as 2^-ΔΔ*C*t value, the reference mRNA being *HPRT* (mean ± SEM., *n* = 2); (**c**) Western blot analysis—Best1 protein is not synthesized by RPE-J or MDCK cells. After transfection, MDCK produce human Best1 at 68 kDa.

*BEST1* was identified as a retina-specific gene expressed in RPE cells [5,6]. Human-derived RPE cell lines ARPE-19 and D407 as well as rat RPE-J cell line express *BEST1* mRNA [11]. To test if MDCK cells express *BEST1* transcript, these cells were investigated with quantitative Real-Time PCR. As shown in Figure 1a, we were able to detect a transcript in MDCK cells expressed at low levels (after 34 cycles of PCR). The level of *BEST1* transcription in MDCK cells was ~60% of the level observed in RPE-J cells (Figure 1b). Although they express *BEST1* mRNA, RPE cell lines do not synthesize the Best1 protein. This is also true for MDCK cells, as they do not produce any Best1 protein either (Figure 1c). MDCK cells transfected with human *BEST1* express Best1 protein at an expected 68-kDa size. On the immunoblot, the upper band with a molecular mass of approximately 70 kDa is considered as a non-specific band as described in RPE-J cells and human RPE using the same antibody in previous studies [7].

### 2.2. Basolateral Localization of Human Best1 in Polarized MDCK Cells

**Figure 2:**
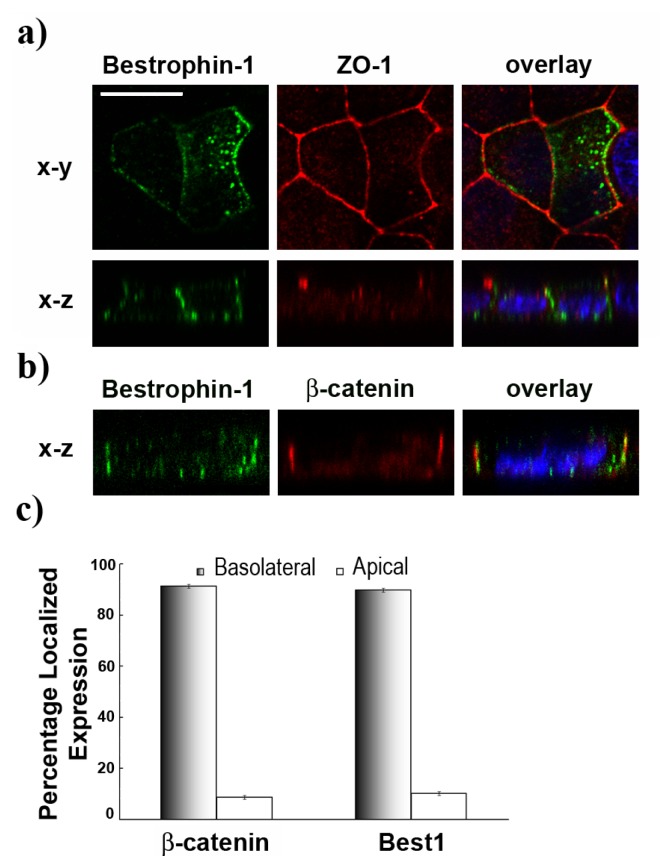
(**a**) Transfected MDCK cells express Best1 (green) at the basolateral surface, colocalizing with the tight-junction marker ZO-1 (red) on X-Y and X-Z single confocal scans; (**b**) Best1 (green) at the basolateral surface, colocalizing with the basolateral marker β-catenin (red) on X-Z single scan. Nuclei are in blue, scale bar = 10 μm; (**c**) Best1 localizes to the basolateral surface at the same level as the well-characterized marker β-catenin. Pixel intensity values obtained from confocal images per Z-focal plane of ten cells transfected with wild type *BEST1* cDNA and the basolateral threshold in each cell were used to calculate percentage localized expression (mean ± SEM., *n* = 10, *p* = 0.018).

*In vivo*, the Best1 protein localizes at the basolateral membrane of RPE cells [11]. In order to test if this localization is similar in MDCK cells, a normal human *BEST1* construct was transiently transfected. The cells were examined by immunofluorescence staining followed by confocal microscopy. Four days after transfection, 5%–10% of cells showed Best1 staining. Development of the epithelial polarity depends on the assembly of tight junctions between the cells [37]. Therefore, cells were investigated with the tight junction marker ZO-1. A normal rim-like morphology typical for polar cells was observed in all cells. In a single transfected cell, X-Y and X-Z confocal cross-section images showed basolateral colocalization of Best1 with ZO-1 (Figure 2a). This basolateral staining was further confirmed by colocalization with β-catenin [38], which reveals membranes separating adjacent cells and on the basal side (Figure 2b). Some intracellular staining was noted, which corresponds to proteins still present in the biosynthetic pathway. Quantification of basolateral and apical signals show similar repartition of Best1 and β-catenin proteins along the different membranes of 10 randomly chosen single cells which do not overexpress Best1 (Figure 2c). Using the trapezoidal calculation approach (see Experimental Section), we show that 90% and 91% of both proteins were preferentially localized to the basolateral membrane, respectively.

### 2.3. Best1 Mutants Are Synthesized by MDCK Cells

**Figure 3:**
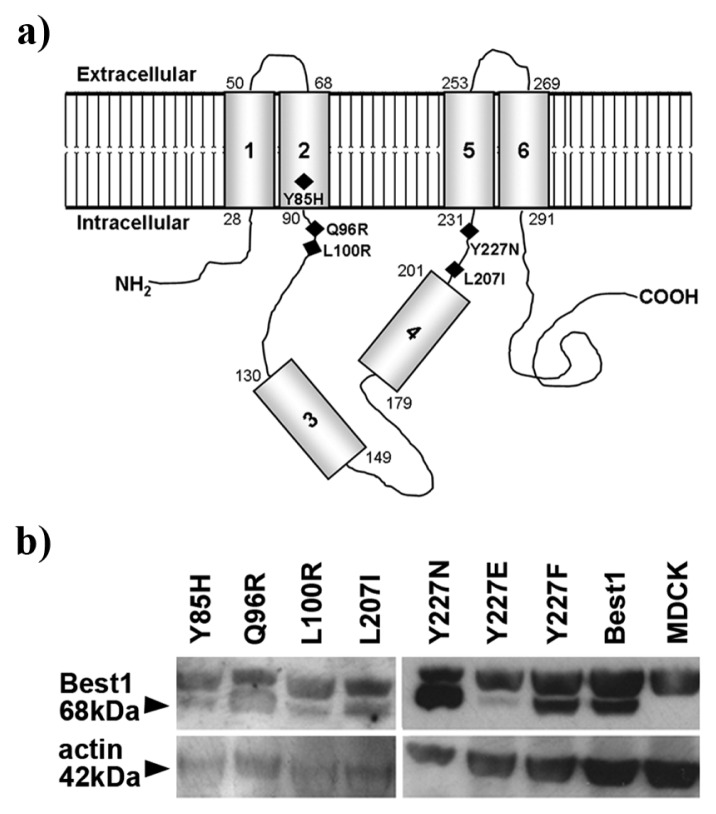
**(a)** Schematic drawing showing localization of the different mutations (black diamonds) tested in our study (modified from [12]); (**b**) Western blot analysis of the normal and mutant human Best1 protein in transiently transfected MDCK cells. Best1 proteins are detectable as a 68 kDa band in all transfected cells, but not in non-transfected controls (MDCK lane). Actin bands are shown to indicate equal loading of cell lysates.

We generated Best1 mutants using site-directed mutagenesis. Mutation sites are localized in putative tyrosine sorting determinants (Y85VTL, Y97ENL, L206L207, Y227DWI) situated in different regions of the protein (Figure 3a). Interestingly, these putative sorting domains correspond to mutations identified in BVMD patients (p.Y85H, p.L100R, p.Y227N). In addition, we included the Q96R mutant, which represents a new mutation identified by our group located next to the Y97ENL motif. Normal and mutant *BEST1* constructs were transiently transfected in MDCK cells. Expression of the different mutants was checked using western blot analysis 48 hours after transfection (Figure 3b). Expression levels of the different forms vary possibly due to different transfection efficiencies or different synthesis rates for each construct tested. For example, expression of mutant Y227E was decreased by 68% (SEM. ± 26, *n* = 2, *p* = 0.016) compared to normal Best1.

### 2.4. BVMD-Related Mutations Affect Best1 Basolateral Localization

**Figure 4:**
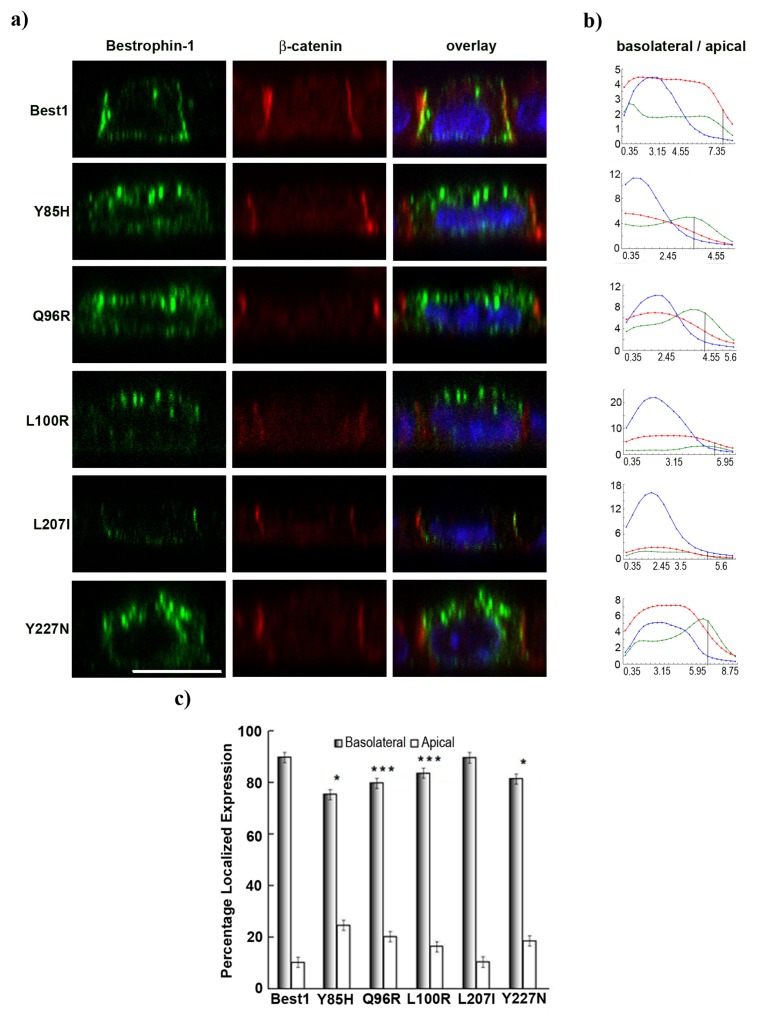
(**a**) X-Z confocal single image scan of transiently transfected cells with different *BEST1* cDNA constructs showing mislocalization of mutants Y85H, Q96R, L100R and Y227N. Cells were stained for Best1 (green), β-catenin (red) and nuclei (blue). Scale bar = 10 μm; (**b**) Z-series confocal stack signals corresponding to each labeling were quantified. Curves indicate the pixel intensity of each section along the Z-axis for each cell (Best1, green; β-catenin, red; nuclei, blue). The black vertical line indicates the Z-focal plane chosen as threshold for apical and basolateral domains separation. Basolateral and apical sides are as indicated. Horizontal axis represents μm distance and vertical axis shows pixel intensities; (**c**) Bar graph illustrating quantification of Best1 mutants distribution in the basolateral and apical domains of the cells compared with normal protein (mean ± SEM., *n* = 10, *p < 0.01, ***p < 0.0001).

To investigate if these mutations influence the localization of Best1, we studied the polarized expression of the different constructs in MDCK cells. X-Z confocal microscopy scans of individual cells showed increased apical staining for mutants Y85H, Q96R, L100R, Y227N when compared to normal Best1 protein (Figure 4a). Pixel intensity values in each Z-focal plane of the same cell indicated different curve shapes for Best1 mutants compared to both β-catenin and normal Best1 (Figure 4b). The peak of maximum intensity for mislocalized Best1 mutants was shifted towards the apical region of the curves, which corroborates well the significant increase in apical staining. Quantitative analyses suggested a significant displacement of Best1 from the basolateral membrane to the apical region at a level of 15% for Y85H (*p* = 5.37 × 10^−5^), 10% for Q96R (*p* = 0.0001), 6% for L100R (*p* = 0.0009) and 9% for Y227N (*p* = 5.20 × 10^−5^) (Figure 4c). In contrast, mutant L207I did not show any change in membrane localization.

**Figure 5:**
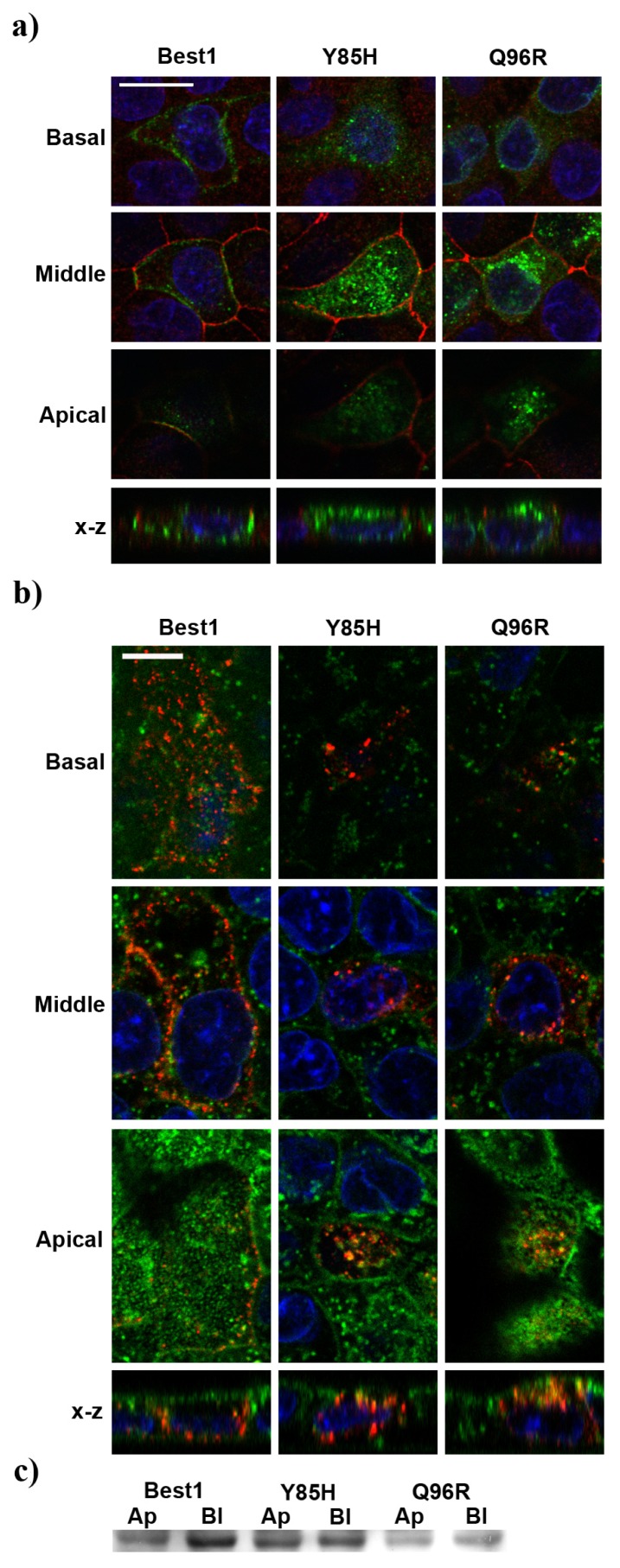
(**a**) Mutant signals (green) are detected above the tight-junction marker ZO-1 (red); (**b**) Mutants (red) show partial colocalization with WGA (green) at the apical membrane (orange signals). Nuclei are in blue. Scale bars = 10 μm; (**c**) Surface-biotinylated Y85H and Q96R proteins confirm increased apical localization (Ap) *versus* basolateral (Bl) localization compared to normal Best1.

We also investigated colocalization of mutants Y85H and Q96R with ZO-1 and wheat germ agglutinin (WGA) as tight-junction and extracellular apical markers respectively. Y85H and Q96R were partially localized apically above the tight junctions of the cells (Figure 5a). We observed partial colocalization of Y85H and Q96R mutants with WGA at the apical membrane that was not detected with normal Best1 (Figure 5b).

Biotinylation assays for the Y85H and Q96R mutants at the apical and basolateral cell membranes show an increase in apical surface localization with respect to wild-type Best1 (Figure 5c).

### 2.5. Pseudo-Phosphorylation of Tyrosine 227 Affects Best1 Basolateral Localization

**Figure 6:**
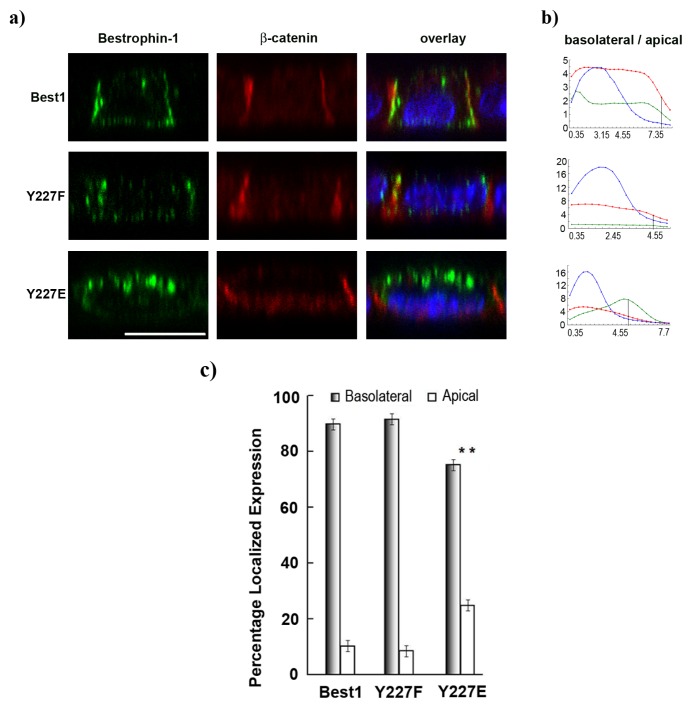
(**a**) X-Z confocal single scan showing that constitutively phosphorylated Best1 (Y227E) is partially targeted to the apical membrane compared to normal and non-phosphorylated (Y227F) Best1. Best1 in green, β-catenin in red, nuclei in blue. Scale bar = 10 μm; (**b**) Quantification of basolateral to apical localization of the three markers (Best1, green; β-catenin, red; nuclei, blue) along the Z-axis confirming the result. Vertical line indicated basolateral-apical domains separation. Horizontal axis represents μm distance and vertical axis shows pixel intensities; (**c**) Bar graph of the repartition percentage of Best1 between basolateral and apical domains (mean ± SEM., *n* = 10, **p < 0.005).

Best1 is a phosphorylated protein [16,39] and activation of drosophila Best1 requires its phosphorylation [40]. As described in the section above, the tyrosine Y227 residue seems important for basolateral sorting. One further question is whether tyrosine-227 phosphorylation is necessary for basolateral targeted expression. In order to address this, polarized sorting of Y227E and Y227F Best1 was analyzed. Pseudo-phosphorylation of tyrosine-227 was performed by substituting tyrosine with glutamic acid, and tyrosine activity was blocked by mutating the tyrosine residue into phenylalanine. Interestingly, constitutively pseudo-phosphorylated Y227E increased apical staining (Figure 6a) and a concomitant peak at the apical region of the quantification curve was observed (+15%, *p* = 0.0013, Figure 6b,c). Non-activated Y227F displayed a basolateral labeling signal and peak in the basolateral side of the quantification curves as is the case for normal Best1. These results suggest a probable role of non-phosphorylated tyrosine-227 for proper basolateral sorting.

Mutations in the *Bestrophin-1 (BEST1)* gene cause a variety of degenerative eye diseases in humans, collectively referred to as “bestrophinopathies” [7,9,41,42]. Best1 protein has been hypothesized to be either a Ca^2+^-activated Cl^−^ channel [43,44], or a regulator of ion transport, or both [14,18,41,42]. In tissues, human *BEST1* mRNA is expressed in the retina/RPE, brain, spinal cord, testis, trachea and kidney [6,45]. In dogs, *Best1* mRNA has been detected in RPE and brain [46], and in mice a wider distribution has been observed [47]. Best1 proteins are localized in the basolateral plasma membrane of RPE cells in macaques, swine [11], humans [28], canines [46] and mice [21,48].

The purpose of this study was to investigate if defective sorting and localization of Best1 protein in polarized epithelial cells could be implicated in the pathogenesis of BVMD. Indeed, Marmorstein and colleagues have shown basolateral sorting of human Best1 by immunofluorescence staining and cell surface biotinylation [11]. Additionally, a study showed leakage of Best1 sorting from the basolateral to the apical membrane of RPE cells in a BVMD patient harboring a p.Y227N mutation in *BEST1* [28]. Milenkovic *et al.* showed disturbed trafficking of some mutant Best1 proteins (including Y227N) in MDCK cells. These cells are a widely used model system to study protein localization in polarized epithelial cells [35]. Unlike RPE-J cells, they grow in a proper monolayer, and basolateral and apical membranes are clearly identified using different markers [49]. Therefore, we decided to use these cells in order to study cellular location of Best1 mutants. First, we analyzed endogenous *BEST1* mRNA and Best1 protein expression. In a number of RPE cell lines (ARPE-19, D407 and RPE-J), *BEST1* transcript can be detected but none of them express the protein endogenously [11]. Similarly, although we detected *BEST1* mRNA after 34 cycles of amplification with Real-Time PCR, Best1 protein was not expressed by native MDCK cells. In contrast, transfected Best1 was expressed at the basolateral membrane of MDCK cells. Thus, we consider that basolateral sorting signals are similarly interpreted by the cell sorting machinery in MDCK and RPE cells, showing that MDCK cells are appropriate for the investigations reported here. Immunofluorescence staining showed a clear and specific signal for normal human and mutant Best1, as well as for β-catenin, ZO-1 and WGA. Our results indicate that human Best1 was preferentially targeted to the basolateral membrane in transfected MDCK cells similarly to the basolateral marker β-catenin.

We examined known and novel *BEST1* mutations, p.Y85H, p.Q96R, p.L100R, p.L207I and p.Y227N, reported as causative for BVMD, some of them also found in our French cohort [6,33,34]. Mutant L207I is preferentially basolaterally localized similarly to normal Best1, suggesting that the putative di-leucine sorting motif L206L207 in the large cytoplasmic domain of Best1 [12] does not seem necessary for basolateral targeting. Isoleucine molecular structure is very similar to leucine structure, and these two amino acids seem to be equally recognized by the cell sorting machinery. In contrast, the other mutants showed altered basolateral sorting. Our colocalization studies with apical and basolateral markers as well as biotinylation data indicate that tyrosine-85, which is a part of the Y85VTL putative sorting motif, also plays a crucial role for basolateral targeting in MDCK cells since mutant Y85H has a decreased basolateral targeting associated with a concomitant increased apical membrane expression compared to normal Best1. The p.Y85H mutation lies in the second transmembrane domain, for which a key role for the tyrosine residue in both the assembly of membrane topology and the conductance of ions across the membrane has been previously demonstrated [12,35]. Therefore, we cannot exclude the hypothesis that impairment of Y85 function would alter membrane targeting due to a different membrane topology instead of the disruption of its putative sorting role. In contrast to strong apical localization shifts observed in confocal images, calculated percentage apical expression changes seem lower because of the choice of highly conservative basolateral/apical thresholds in each cell. We also observed an altered basolateral to apical sorting in mutants Q96R and L100R, sitting near the tyrosine sequence Y97ENL in the large cytoplasmic loop of Best1 [12], another putative sorting motif. Since the expression of both mutants is apically increased, we speculate this may be due to the altered exposure of the tyrosine motif to the cell sorting environment compared to the native protein. Using surface biotinylation assays, we confirmed that synthesized Y85H and Q96R proteins are delivered to the plasma membrane. Although, it is also possible that some amounts of the protein are retained in the cytoplasm.

Tyrosine-227 marks another putative sorting motif Y227DWI, and in a BVMD patient the p.Y227N mutation leads to partial mistargeted apical distribution of the protein in the RPE [28]. In accordance with this, we found lower basolateral expression of mutant Y227N (81%) in polarized MDCK cells. Mislocalization of Y227N and D228N human Best1 has been reported [8,36], confirming the importance of the Y227DWI putative sorting motif for Best1 basolateral expression. In addition, a significantly lower percentage of the active Y227E variant is found at the basolateral membrane domain in MDCK cells in contrast to the inactive Y227F mutant and normal Best1 (75% *versus* 92% and 90%, respectively). We speculate and attribute the altered localization of the proteins to constitutive pseudo-phosphorylation of tyrosine-227 (Y227E), suggesting that it is important to keep tyrosine-227 non-phosphorylated (Y227F) for correct basolateral localization of Best1. Y227E and Y227N mutants both comprise hydrophilic charges that might influence the protein localization due to “pseudo-phosphorylation”-like effect. Into the Best1 molecule there are several tyrosine residues that could be phosphorylated, and direct proof for specific phosphorylation at Y227 would help us support or exclude such hypothesis. Another hypothesis would be that these amino acid modifications (asparagine and glutamate have similar structures) might influence the tridimensional structure of the protein, thus impairing its proper membrane targeting. In any case, it is clear from our data and from other published work [8,28,36] that the Y227DWI motif is important for proper Best1 directing at the basolateral membrane.

Our findings indicate an important role of some amino acids into and close to the putative sorting tyrosine motifs Y85VTL, Y97ENL, Y227DWI for basolateral expression of Best1. Y85VTL and Y97ENL motifs are closer to each other compared to Y227DWI, which is almost at the end of the large cytoplasmic domain. Potentially, they might convey an accumulative effect on polarized sorting, which has not been investigated in this study. If the mutant proteins are mistargeted to the apical membrane, they would be between 60% and 150% more abundant than the normal protein at that domain. We therefore hypothesize that this altered basolateral localization of Best1 could be responsible for altering the ion conductance both at the basolateral and the apical membranes of the cells. The diminished basolateral presence of Best1 proteins might explain the decrease of the light peak as measured by EOG in BVMD patients carrying these particular *BEST1* mutations [21]. However, other pathogenic mechanisms than protein mislocalization exist, as suggested by the discrepancy between the absence of localization phenotype we observed for mutant L207I that gives rise to BVMD pathology in patients.

Our results show mislocalization of Best mutants disrupting potential sorting motifs Y85VTL, Y97ENL and Y227DWI. Importantly, tyrosine motifs need to be located in the intracellular portion of the protein to be recognized by the cell sorting machinery [29,31]. If we refer to the model by Milenkovic and colleagues (see Figure 3a), most tyrosine motifs we discussed are displayed in the cytoplasm of the cell where they are available for recognition. In contrast, the model by Tsunenari and colleagues assumes that TMD4 traverses the membrane while TMD5 is inserted in the extracellular side of the lipid bilayer, parallel to the surface [13]. This Best1 tertiary structure places both important motifs Y97ENL and Y227DWI inside the lipid bilayer and outside of the cell respectively, where they cannot be recognized by the cell machinery. According to the Milenkovic model, the localization shift of our mutants seems to be proportional to the proximity of the mutation to the cell membrane (e.g., Y85H > Q96R-Y227N > L100R). Taken together, we consider our findings fit better the Best1 model structure proposed by Milenkovic and colleagues, where TMD4 is part of a hydrophobic intracellular loop and TMD5 is an integral transmembrane domain [12].

The function of Best1 is still controversial and incompletely understood, but it is clear that the proper localization of the protein is crucial for homeostasis and function of RPE and retina. Observations in this report suggest that altered basolateral localization of Best1 protein may participate in the disease mechanism in BVMD patients. Polarized sorting of Best1 is a complex mechanism that may require several sorting motifs and the phosphorylation state of some tyrosine residues could be part of the sorting process.

## 3. Experimental Section

### 3.1. Materials

All reagents and chemicals were supplied by Sigma-Aldrich (St. Quentin Fallavier, France) unless otherwise stated. DNA ladder was obtained from Biolabs (Seville, Spain) and Amersham High-Range Rainbow Molecular Weight Marker from GE Healthcare (Barcelona, Spain), DAPI from Invitrogen (Cergy-Pontoise, France), Polymount mounting buffer from Biovalley (Conches, France). EZ-Link™ sulfo-*N*-hydroxy-succinimidyl-biotin (Sulfo-NHS-biotin) and the streptavidin-horseradish peroxidase (HRP) conjugate were obtained from Pierce (Madrid, Spain). Pansorbin^®^ cells were supplied by Calbiochem (Madrid, Spain). Antibodies were obtained as follows: mouse IgG1 antibody (E6-6) directed against the *C*-terminal domain of human Best1 from Novus Biologicals Inc. (Littleton, France) [7,20,50], rabbit whole antiserum β-catenin antibody and rabbit IgG antibody against actin from Sigma-Aldrich, rabbit anti-ZO-1 from Zymed (Invitrogen, Cergy-Pontoise, France), HRP-linked anti-mouse and anti-rabbit IgG from Amersham Pharmacia Biotech (GE Healthcare, Barcelona, Spain), goat anti-mouse AlexaFluor 488 and 594, goat anti-rabbit AlexaFluor 633 from Invitrogen, and FITC-conjugated wheat germ agglutinin (Sigma, St. Quentin Fallavier, France).

### 3.2. Expression Vector and Mutagenesis

The cDNA sequence of human *BEST1* gene cloned into pReceiver vector was purchased from imaGenes GmbH (Berlin, Germany). The ORF contains 1758 nucleotides, a TAG stop codon and four known synonymous polymorphisms: c.109T > C, p.L37L; c.219C > A, p. I73I; c.1410G > A, p.T470T; c.1608T > C, p.T536T with NCBI SNP reference numbers rs1800007, rs1109748, rs149698 and rs5835779, respectively. The vector contains ampicillin and neomycin resistance genes. Gene expression is under the control of the CMV promoter.

All *BEST1* mutants—p.Y85H (c.253T > C); p.Q96R (c.287A > G); p.L100R (c.299T > G); p.L207I (c.619C > A); p.Y227N (c.679T > A); p.Y227E (c.679T>G and c.681C > G) and p.Y227F (c.680A > T) were generated using QuickChange® II Site-Directed Mutagenesis Kit (Stratagene, Massy, France) and were confirmed via direct sequencing. The primers were chosen using QuickChange^®^ Primer Design Program (Stratagene, Les Ulis, France) (Table 1) and purchased from Invitrogen (Cergy-Pontoise, France).

### 3.3. Real-Time PCR Analysis

Total RNA was extracted using TRIzol extraction kit (Invitrogen, Cergy-Pontoise, France) according to the manufacturer’s instructions. The QuantiTect Reverse Transcription Kit (Qiagen, Seville, Spain) was used to generate cDNAs from 1 μg of total RNA in a 40-μL total reaction volume. Primers were designed within the coding sequence and spanning introns (Sigma-Aldrich, St. Quentin Fallavier, France). We used the hypoxanthine phosphoribosyltransferase (*HPRT*) as reference mRNA. Primer sequences used for analysis in RPE-J cells were *HPRT* 5′-GTTCTTTGCTGACCTGCTGG-3′, 5′-GTTGAGAGATCATCTCCACC-3′, and *BEST1* 5′-GTGTGCACCTTGCGTACTC-3′,5′-GTAGTTCTTG AGTGGGTTCAG-3′. Primers sequences spanning the same amplicon used for analysis in MDCK cells were *HPRT* 5′-CCTCATGGACTAATTATGGAC-3′, 5′-CTGTTCAGTGCTTTGATATAATC-3′, and *BEST1* 5′-CGATGGAGCGGGATATGTAC-3′,5′-GTGCCTTCCTCTTCCTCCT-3′. Real-Time PCR was carried out using the 7500 Fast Real-Time PCR machine (Applied Biosystems, Madrid, Spain). The PCR reaction was performed using 1 μL cDNA, 10 μL SYBR Green master mix (Applied Biosystems, Cergy-Pontoise, France) and 400 nM primer in a total reaction volume of 20 μL. PCR conditions were as follows: 50 °C for 2 min and 95 °C for 10 min; 40 cycles at 95 °C for 15 s and 60 °C for 1 min. We repeated the experiments twice, and in every experiment each sample was amplified in triplicates. Both Real-Time PCR products have been confirmed via direct sequencing. Differences between the mean cycle threshold (*C*t) values of *BEST1* and *HPRT* genes were calculated as Δ*C*t_BEST1_ = *C*t_BEST1_ − *C*t _HPRT_. Relative fold change in *BEST1* expression levels was determined as 2^−ΔΔ^*^C^*^t^, and we considered *BEST1* expression in MDCK cells in comparison with RPE-J by calculating 2^−ΔΔ^*^C^*^t^ = Δ*C*t_MDCK_ − Δ*C*t_RPE-J_.

### 3.4. Cell Culture

MDCK cells (ATCC, Teddington, UK), strain II, were grown in DMEM with 10% FCS, streptomycin (100 mg/L) and penicillin (60 mg/L) at 37 °C and 5% CO_2_. RPE-J cells [51] were maintained in DMEM with 4% FCS, 1% non-essential amino acids, 1% HEPES, streptomycin (100 mg/L) and penicillin (60 mg/L) at 32 °C and 5% CO_2_. For polarity studies, 30,000 and 200,000 MDCK cells were plated and grown on coverslips in 24-well plates and on 24-mm (6-well plates) Costar Transwell filters (Corning, Sofia, Bulgaria) for five days, respectively [52,53]. The state of polarity was confirmed by measuring the transepithelial resistance (data not shown). For cell extraction studies, 30,000 MDCK and RPE-J cells were plated and grown in 24-well plates for 3 days. Transfections of MDCK cells were performed 24 h after cell seeding using the Effectene^®^ Transfection Reagent (Qiagen, Sofia, Bulgaria).

### 3.5. Cell Lysis and Biotinylation Assay

Cells were extracted with 200 μL RIPA buffer and a cocktail of protease inhibitors 48 h after transfection. 100 μL of cell lysates were precipitated with acetone at −20 °C. Precipitates were then dissolved in reducing sample buffer and separated on 10% SDS-PAGE gels.

Transiently transfected MDCK cells were washed three times with PBS^++^ (PBS with 0.1 mM CaCl_2_ and 1 mM MgCl_2_) and once with a biotinylation buffer (120 mM NaCl, 20 mM NaHCO_3_, 1 mM MgCl_2_, 0.1 mM CaCl_2_, pH 8.5) at 4 °C for 15 min. Sulfo-NHS-biotin labeling (0.5 mg/mL in biotinylation buffer) was performed twice for 20 min at 4 °C either basolaterally or apically with 1% BSA in PBS^++^ on the opposite site. For each transfection with Best1 mutants Y85H and Q96R, three wells for apical and three for basolateral labeling were used. Cells were washed once with DMEM supplemented with 0.2% BSA and 20 mM Hepes, pH 7.4, and three times with PBS^++^ for 5 min at 4 °C [52–54].

### 3.6. Immunoprecipitation

Cells were washed twice with PBS^++^ and solubilized in 0.5 mL lysis buffer (1% IGEPAL^®^ CA-630 (Sofia, Bulgaria), 10 mM Tris-HCl pH 7.4, 60 mM EDTA, 0.4% deoxycholic acid) in the presence of protease inhibitors (0.75 mM PMSF, 10 μg/mL aprotinin, 10 μg/mL leupeptin and 10 μg/mL pepstatin) for 30 minutes at 4 °C. Lysates from triplicates for each transfection and labeling were collected together. Insoluble materials were removed by centrifugation (13,000 rpm, 5 min) and supernatants were pretreated with Pansorbin^®^ cells for 1 h at 4 °C. SDS was added to a final concentration of 0.3%. After overnight incubation at 4 °C with a human anti-Best1-specific mouse antibody (IgG1, clone E6-6) immune complexes were precipitated with Protein G-sepharose (5 mg/mL washing buffer, 1% IGEPAL^®^ CA-630 (Sofia, Bulgaria), 0.5% deoxycholic acid, 0.1% SDS, 150 mM NaCl, 10 mM Tris-HCl pH 7.4). After centrifugation (13,000 rpm, 1 min) the sepharose beads were washed four times with washing buffer and boiled in reducing sample buffer. The eluted proteins were separated by 10% SDS-PAGE [52,53].

### 3.7. Immunoblotting

Electrophoretically separated proteins were transferred to PVDF membranes (GE Healthcare, Sofia, Bulgaria) by wet western blotting. Non-specific binding was blocked with 10% BSA in TBS for 1 hour. Blots were incubated overnight with human anti-Best1*-*specific mouse antibody at 4 °C followed by incubation with HRP-conjugated anti-mouse IgG antibody for 2 h at room temperature. To detect actin proteins, membranes were incubated with rabbit IgG antibody against actin followed by HRP-conjugated anti-rabbit IgG antibody. For detection of biotinylated Best1 and mutant proteins, non-specific binding was blocked with 5% BSA in TBS-N for 1 h, and blots were incubated with HRP-conjugated streptavidin in TBS-N for 1 h. Immunoreactivity was detected using the ECL plus western blotting detection system (GE Healthcare, Sofia, Bulgaria) according to manufacturer’s instructions. Bands were detected using Amersham Hyperfilm™ MP (GE Healthcare), films were scanned and sample signals from the same experiment present on the same blot were quantified with NIH ImageJ 1.42 software (version 1.42, NIH, Bethesda, MD, USA). We calculated Best1/actin ratios for each experiment, calculated mean ratios and SEM., and tested for significance with Student’s *t*-test.

### 3.8. Immunofluorescence Staining and Microscopy Analysis

Transiently transfected MDCK cells expressing human Best1 or Y85H, Q96R, L100R, L207I, Y227N, Y227E and Y227F mutants were grown for five days on coverslips and fixed with ice-cold methanol. After blocking with PBS^++^ containing 1% BSA for one hour, cells were incubated with a mouse anti-Best1, rabbit β-catenin, rabbit ZO-1 and WGA overnight at 4 °C. Bound antibodies were detected using proper secondary antibodies for two hours at room temperature. For negative controls, samples were incubated with the secondary antibody alone. Nuclei were labeled with DAPI. Best1 and WGA fluorescence was visualized using an Olympus FV1000 microscope and Fluoview 2.0 software (version 2.0, Olympus, Rungis, France), with a 60× objective, 4-time zoom and 0.45-μm step size scans. For all confocal data used for quantification, we acquired images using Leica TCS SP5 confocal microscope and LAS AF software (version 2.6.1, Leica Microsystems, Wetzlar, Germany), with a HCX PL APO lambda blue 63.0 × 1.40 OIL objective and 4-time zoom enlargement. Z- and Y-series image data were acquired in 0.35- and 0.30-μm step size sequential scanning respectively. Series data were analyzed using METAMORPH 7.5.1.0 software (version 7.5.1.0, Molecular Devices, Sunnyvale, CA, USA). Signals for Best1, β-catenin (basolateral marker) [38] and nuclei were quantified. Image stacks were used to generate intensity curves for all three signals along the different Z-focal planes. Ten randomly chosen cells from normal Best1 and each mutant were analyzed. The number of focal planes varies in the different cells depending on the slightly different cell height. Basolateral localization threshold values were defined by visual exploration of confocal images and by examination of Best1 and β-catenin curves for each cell where 90% of β-catenin is basolateraly sorted. After detection of basolateral thresholds, the area under the Best1 curve was calculated for basolateral and apical portions of localization for each cell, respectively. The trapezoidal method is used to approximate the area under the bestrophin-1 curve, by inscribing or circumscribing “*n*” number of trapezoids under a curve. Areas of the trapezoids are then summed for basolateral and apical region of the curve respectively. The number of trapezoids under the curve is equal to the number of confocal plane points represented by the curve. *p* values were computed using two-sided independent sample Student’s *t*-tests, comparing the bestrophin-1 localization for each mutant to the normal protein.

## 4. Conclusions

Alterations of the cellular localization of Best1 mutants may affect the ion equilibrium and overall functioning of the RPE cells representing an interesting insight into the underlying pathogenic mechanisms of BVMD. Our data indicate that several disease-causing mutations are associated with altered localization of hBest1 protein. As well, at least three putative basolateral sorting motifs might be implicated in proper Best1 basolateral localization. In addition, non-phosphorylated tyrosine 227 may play a role in basolateral delivery.

## Figures and Tables

**Table 1:** Primers used for site-directed mutagenesis.

Mutants	Primers
Best1 Y85H	F 5′-CGTGCTGGGCTTCCACGTGACGCTGGT-3′ R 5′-ACCAGCGTCACGTGGAAGCCCAGCACG-3′
Best1 Q96R	F 5′-CCGCTGGTGGAACCGGTACGAGAACCTGC-3′ R 5′-GCAGGTTCTCGTACCGGTTCCACCAGCGG-3′
Best1 L100R	F 5′-CAGTACGAGAACCGGCCGTGGCCCGAC-3′ R 5′-GTCGGGCCACGGCCGGTTCTCGTACTG-3′
Best1 L207I	F 5′-GGGACCCTATCCTGATCCAGAGCCTGCTG-3′ R 5′-CAGCAGGCTCTGGATCAGGATAGGGTCCC-3′
Best1 Y227N	F 5′-GTGGACACCTGTATGCCAACGACTGGATTAGTATC-3′ R 5′-GATACTAATCCAGTCGTTGGCATACAGGTGTCCAC-3′
Best1 Y227E	F 5′-GTGGACACCTGTATGCCGAGGACTGGATTAGTATCCC-3′ R 5′-GGGATACTAATCCAGTCCTCGGCATACAGGTGTCCAC-3′
Best1 Y227F	F 5′-GTGTGGACACCTGTATGCCTTCGACTGGATTAGT-3′ R 5′-ACTAATCCAGTCGAAGGCATACAGGTGTCCACAC-3′
